# Effects of sodium alginate active films containing different lotus rhizome root powders on physicochemical properties and shelf-life of pork patties

**DOI:** 10.5713/ab.25.0028

**Published:** 2025-05-12

**Authors:** Zhuangzhuang Qiu, Koo Bok Chin

**Affiliations:** 1 School of Public Health, Jining Medical University, Jining, China; 2 Department of Animal Science, Chonnam National University, Gwangju, Korea

**Keywords:** Lotus Rhizome Root Powder, Physicochemical Properties, Pork Patty, Shelf-life, Sodium Alginate Active Film

## Abstract

**Objective:**

This study was done to investigate the film properties and antioxidant activities of sodium alginate films (SAFs) added with different levels (0.5, 1, and 2%) of oven-dried (100°C) lotus rhizome root powder (ODLRRP).

**Methods:**

After pork patties were wrapped with different SAFs, the physiochemical properties and antimicrobial and antioxidant activities of pork patties were determined.

**Results:**

SAFs containing ODLRRP decreased the pH, lightness (L*), and equilibrium water content but increased the redness (a*), yellowness (b*), transparency, moisture content, and antioxidant activities. SAFs containing ODLRRP increased the color values, but L*, 2-thiobarbituric acid-reactive substances, microbial counts, and water content decreased. In pork patties wrapped with SAFs containing ODLRRP at levels greater than 1% ODLRRP, the L*, volatile basic nitrogen, and total bacterial counts decreased, whereas b* increased.

**Conclusion:**

These results indicated that pork patties wrapped with SAFs containing more than 1% ODLRRP could inhibit microbial growth and reduce protein denaturation.

## INTRODUCTION

Renewable resources, such as alginate films, are being explored to generate biodegradable materials owing to the unique film-forming properties of alginate [1,2]. Food quality affects consumer acceptance and is determined by its odor, flavor, texture, and color [3,4]. Tavassoli-Kafrani et al [5] reported that edible films and coatings derived from alginate could be incorporated with food additives to extend the shelf-life of meat or meat products, thus extending their application in the food industry.

Qiu and Chin [6] reported that regular-fat sausages wrapped with sodium alginate films (SAFs) containing cherry tomato powder showed increased antimicrobial and antioxidant activities. Whey protein-chitosan films inhibited microbial growth and pathogenesis in fresh turkey pieces [7]. Edible films containing soy protein supplemented with oregano and thyme showed reduced microbial counts of *Pseudomonas* spp. and coliform bacteria in beef patties [8]. Results of increased shelf-life due to films or coatings prompted us to use oven-dried lotus rhizome root powder (ODLRRP) in SAFs for wrapping pork patties.

Lotus rhizome root (LRR) growing in fresh water is an edible and delicious food containing many polyphenols, which contribute to the browning reaction [9]. The lotus root contains many flavonoids and phenolic compounds with antioxidant and antibacterial activities [10,11]. Ham et al [12] reported that the 2-thiobarbituric acid-reactive substances (TBARS) in pork sausages added with lotus rhizome root powder (LRRP) was lower than that in control (untreated) samples, which might be attributed to the antioxidant activity of phenolic compounds in LRRP. Shin et al [13] reported that the TBARS value of pork patties supplemented with lotus root extract was lower than that of control samples and that patties added with lotus leaf extract showed the lowest TBARS value among all treatments. These findings demonstrated that LRRP and lotus root extract improved the antioxidant properties of sausages and patties. However, the effects of LRRP films or coatings on meat products, such as sausage, have not been studied yet. Therefore, the objective of the present study was to investigate the film properties and antioxidant activities of SAFs added with different levels (0.5%, 1.0%, and 2.0%) of LRRP oven-dried at 100°C (ODLRRP). After pork patties were wrapped with SAFs containing various levels of ODLRRP, the physicochemical properties and antimicrobial and antioxidant activities of both SAFs and pork patties were determined.

## MATERIAL AND METHODS

### Preparation of lotus rhizome root powder, pork patties, and sodium alginate films

LRR was purchased, washed, and chopped into slices (3 cm). A drying oven (LDO-250F; Labtech, Ltd., Jeonju, Korea) was used to dry these slices at 100°C for 8 h according to the method described by Kim and Chin [14]. Dried slices were blended into a powder, sieved to particle sizes below 150 μm, and stored at −70°C before use.

Pork hams and back fat were purchased from a local meat market of Gwangju. They were trimmed and ground using a grinder (M-12s; Fujee Plant, Busan, Korea). The mixture of meat batter, sodium chloride, and ODLRRP was reground to form patties, each containing approximately 30 g of the mixture. Pork patties were placed in a silica-polypropylene box and stored at 4±1°C for 14 days.

Films were made according to our previous report [6] with slight modifications. Briefly, dried LRRP was mixed with 2% sodium alginate and glycerol. Approximately 18 g of each solution was poured evenly onto a glass agar plate and dried in the drying oven at 50°C for 24 h. Subsequently, 50 mL of 2% calcium chloride solution was added to cross-link each film for 30 s. The cross-linked films were removed from the agar plate and dried at room temperature for about 6 h.

### Properties of sodium alginate films with different concentrations of oven-dried lotus rhizome root powder

#### pH values of film mixture solutions

A pH meter (Mettle-Toledo, Schwarzenbach, Switzerland) was used to measure the pH of each film mixture solution five times.

#### Weight increase of sodium alginate films after cross-linking

The initial weight (W_0_) and cross-linked weight (W_1_) of each film were recorded. The weight increase (WI, %) was calculated using the following formula: WI = (W_1_−W_0_)×100/W_0_.

#### Thickness

A digital center distance caliper (5118-150; Yangzhou Goldenwell Import & Export Co., Ltd., Jiangsu, China) was used to measure the thickness (X_1_) of each film using the method of Siripatrawan and Harte [3] with a slight modification.

#### Light transmission

Each film was cut into a rectangle, and a spectrophotometer (UV-1601; Shimadzu, Kyoto, Japan) at a wavelength of 600 nm was used to measure the absorbance (Abs_600_). The transparency was measured as Abs_600_ /X_1_, where X_1_ was the thickness (mm) of the film. A higher value indicated a lower transparency and a higher degree of opacity.

#### Color values

Color values of films were measured using the method of Rhim [2] with a slight modification. A color reader (Model CR-10; Minolta, Tokyo, Japan) was used to measure the lightness (L*), redness (a*), and yellowness (b*) of each film as follows: ΔL = L*_std_−L*, Δa = a*_std_−a*, Δb = b*_std_−b*, where L*_std_ = 94.7, a*_std_ = 3.6, and b*_std_ = −10.6.


$$\text{Total color difference }\left(\Delta E^{*}\right)=\sqrt{\Delta {\text{L}}^{2}+{\text{a}}^{2}+{\text{b}}^{2}},\;\text{Chroma }\left(C^{*}\right)=\sqrt{{\text{a}}^{2}+{\text{b}}^{2}}.$$

#### Moisture content and water solubility

A filter paper thimble containing 0.5 g of each film was oven-dried at 100°C for 24 h to measure the weight (W_1_). Moisture content (MC, %) was calculated as follows: MC (%) = (0.5−W_1_)×100/0.5.

Each dried film (0.2 g) was soaked in 10 mL of double-distilled water (dd-H_2_O) and stored at 4°C for 24 h [2]. The undissolved film was dried at 100°C for 24 h (W_1_) to determine the water solubility (WS) using the following formula: WS = (0.2−W_1_)×100/0.2.

#### Swelling ratio and equilibrium water content

The swelling ratio (SR) was determined using the method of Rhim [2] with a slight modification. The initial weight of each film (W_0_) was approximately 0.2 g. The weight after dipping in 20 mL of dd-H_2_O (W) was determined after storing at 4°C for 20, 40, 60, 80, and 100 min. The SR was calculated as follows: SR = (W−W_0_)/W_0_. The equilibrium water content (EWC) was determined using the following formula: EWC = (W_e_−W_0_)/W_e_, where W_e_ was the swollen film weight at equilibrium, and W_0_ was the initial weight of the film.

#### Antioxidant activities of films

Each film sample was dissolved in 15 mL of dd-H_2_O to obtain levels of 0.5%, 1.0%, and 2.0%. The antioxidant activity was determined by measuring the total phenolic compounds (TPCs), 2,2-diphenyl-1-picrylhydrazyl (DPPH) radical-scavenging activity, ferrous iron-chelating ability (FICA), and ferric reducing power ability (FRPA) of each film incorporated with different amounts of ODLRRP based on the study of Kim and Chin [14]. TPCs were measured by treating each 2% film solution (100 μL) with dd-H_2_O (2.9 mL), sodium carbonate (2%, 2 mL), and Folin–Ciocalteu reagent (50%, 100 μL) before measuring the absorbance at 750 nm. Each film solution (1 mL) was mixed with 0.25 mL of methanolic DPPH radical solution (0.2 mmol L^−1^) and left in the dark for 30 min before measuring the absorbance at a wavelength of 517 nm. To determine FICA, 0.5 mL of each film solution was mixed with 100 μL of ferrous chloride (0.6 mmol L^−1^) and then treated with 1 mL of methanol or 0.9 mL of methanol and 100 μL of ferrozine (5 mmol L^−1^) before incubating at room temperature for 5 to 10 min, followed by measurement of absorbance at 562 nm. FRPA was determined by mixing 2.5 mL of each film solution with 2.5 mL of 0.2 mmol L^−1^ sodium phosphate buffer (pH 6.6) and 2.5 mL of 10 mg mL^−1^ potassium ferricyanide. Each mixture was then treated with 2.5 mL of trichloroacetic acid (100 mg mL^−1^) after incubating in a drying oven at 50°C, followed by centrifugation at 670×g for 10 min. Finally, 2.5 mL of each supernatant was mixed with 2.5 mL of dd-H_2_O and 0.5 mL of ferric chloride (1 mg mL^−1^) or 3 mL of dd-H_2_O and left at room temperature for 10 min before measuring its absorbance at 700 nm.

#### 2-Thiobarbituric acid-reactive substances

The TBARS were measured using the method of Sinnhuber and Yu [15] with minor modifications. Briefly, each film (2 g) was mixed with 2.5% trichloroacetic acid (3 mL) and 1% thiobarbituric acid (17 mL). The mixture was boiled at 90°C for 30 min. Then, 5 mL of the supernatant was dissolved uniformly with 5 mL chloroform and centrifuged at 670×g for 5 min. Then, 3 mL of petroleum ether and 3 mL of supernatant were mixed and centrifuged at 670×g for 10 min. The absorbance of the bottom layer was measured at 532 nm using a spectrophotometer.

#### Volatile basic nitrogen

The volatile basic nitrogen (VBN) contents (mg%) in films were determined using the method of Li et al [16]. First, 1 g of each film and 9 mL of dd-H_2_O were homogenized for 1 min. Then, 1 mL of filtrate was obtained using a Whatman No. 1 filter and reacted with 1 mL of 50% potassium carbonate solution in a Conway dish. The middle portion of the Conway dish was treated with 1 mL of boric acid (0.01N) and three drops of VBN indicator, followed by incubation in a dry oven at 37°C for 2 h. The resultant solution was titrated with 0.01N HCl until a red color was obtained.

#### Microbial counts

Sterilized water (90 mL) was used to dilute 10 g of each sample. Then, 0.1 mL of the mixture was evenly spread onto a total plate count (TPC) or violet red bile (VRB) agar plate and incubated at 37°C for 24 h. Colony counts of total bacteria (TBC) and *Enterobacteriaceae* were recorded from TPC and VRB agar plates, respectively, and expressed as log CFU/g.

#### Increase in water (%)

The initial weight of SAF (W_0_) and the storage weight of SAF (W_s_) were recorded. The increase in water (IW) was calculated as (W_s_−W_0_)/W_0_.

### Physicochemical and textural properties of patties wrapped with sodium alginate films incorporated with different concentrations of oven-dried lotus rhizome root powder

#### pH, color values (L*a*b*), and proximate compositions of pork patties

A pH meter (Mettle-Toledo) was used to evaluate each patty five times. The color values (L*a*b*) of each pork patty were measured with the Minolta CR-25 color reader six times. The MC (%), crude fat content (%), and crude protein content (%) for proximate compositions were measured using the method of the Association of Official Analytical Chemists [17]. The MC was measured based on weight difference using a drying oven at 100°C for 16–24 h. Crude fat content (%) was determined using the Soxhlet fat extraction method. Crude protein content (%) was determined with a steam distillation unit (Kjeldahl Semi-Automatic Pro-Nitro S 4002851; Selecta Co., Ltd., Abrera, Spain).

#### 2-Thiobarbituric acid-reactive substances, volatile basic nitrogen contents, and microbial counts of pork patties

The TBARS, VBN contents, and microbial counts of pork patties were determined following the methods described in sections 2.2.9, 2.2.10, and 2.2.11 with slight modifications, respectively.

#### Weight loss

The weight loss (WL, %) of the pork patties during the storage period was determined by measuring the weight difference before and after removing the SAF.

### Statistical analysis

Film properties were analyzed by one-way analysis of variance (ANOVA) using the Windows 21.0 program. DPPH, FICA, and FRPA results of antioxidant activities in different films were analyzed by two-way ANOVA using film concentrations and treatments as main factors, whereas TPCs of films were analyzed by one-way ANOVA. Pork patties subjected to different treatments and storage days were analyzed by two-way ANOVA. Each replication was a random effect. The whole experiment was repeated three times. Significant differences were determined using Duncan’s multiple range test at p<0.05.

## RESULTS AND DISCUSSION

### Film properties and antioxidant activity of different sodium alginate films

#### pH, WI, thickness, transparency, color values (L*, a*, b*), moisture content, water solubility, swelling ratio, and equilibrium water content

The physicochemical properties of different SAFs are listed in Table 1. The pH values of SAF solutions added with ODLRRP were lower than those of SAF solutions without ODLRRP (p<0.05). The pH values of SAFs incorporated with 1% ODLRRP were lower than those of SAFs added with 0.5% ODLRRP (p<0.05). This was partially due to the low pH (5.7) of ODLRRP itself. In the present study, oven drying triggered the Maillard reaction, resulting in the formation of organic acids, which might reduce the pH value of ODLRRP and affect the shelf-life [18]. Brands and Van Boekel [18] showed that the organic acids formed due to the Maillard reaction of the monosaccharide-casein system during heating affected the decrease in the pH value.

In the present study, there was no difference in the WI of different level of SAFs after cross-linking (Table 1). The thickness of SAFs was also not affected by the ODLRRP addition. Higher values of absorbance indicated a lower transparency and a higher degree of opacity, indicating that the transparency of SAFs decreased with increasing addition of ODLRRP, due to a higher opacity value (p<0.05). High transparency of food packaging or coating is appreciated by consumers [19]. However, in the present study, the addition of ODLRRP decreased the transparency of SAF, which might be attributed to the incompatibility between ODLRRP and SAF solution. In a related study, Yoo and Krochta [19] reported that blended films consisting of whey protein isolate (WPI) and hydroxypropyl methylcellulose (HPMC) or sodium alginate had lower transparency than those of films containing WPI, HPMC, and sodium alginate alone.

The L* value of SAFs tended to decrease with the addition of ODLRRP. It further decreased with increasing addition of ODLRRP, whereas the values of a*, b*, ΔE*, and C* tended to increase with increasing addition of ODLRRP. These changes in the color of SAFs were mainly due to the inclusion of the color of ODLRRP, which was reddish-brown.

The addition of ODLRRP did not affect the MC and WS of SAFs (p>0.05). However, the SR of SAFs was decreased by the addition of ODLRRP (data not shown). SR tended to increase from 0 to 60 min followed by decreases between 80 to 100 min. Li et al [20] reported that the SR of polyacrylamide films increased with enhanced solvent quality and decreasing cross-linking. Under the water equilibrium condition of the system, the water activity (*a*_w_) remained constant [21]. The EWC of SAFs was determined during 60 to 80 min, showing a decrease with the addition of ODLRRP, which affected the *a*_w_ of SAFs.

#### Total phenolic compounds, DPPH, ferrous iron-chelating ability, and ferric reducing power ability

**Figure 1:**
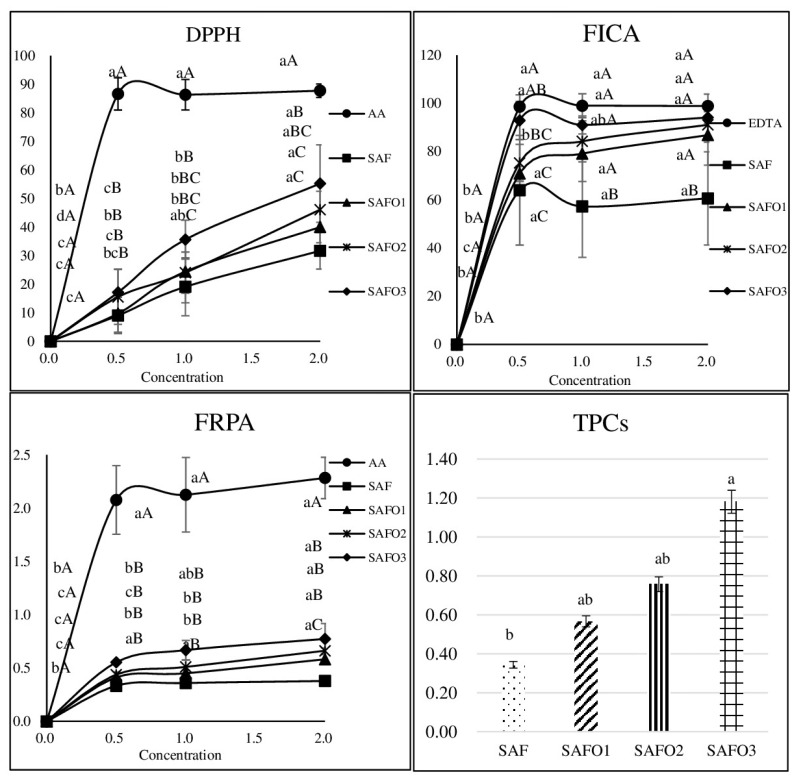
Antioxidant activities of SAFs incorporated with different levels of ODLRRP. ^a–c^ Different superscripts in the same treatment are significantly different (p<0.05). ^A–C^ Different superscripts in the same concentration are significantly different (p<0.05). DPPH, radical-scavenging activity; FICA, ferrous iron-chelating ability; FRPA, ferric reducing power ability; TPC, total plate count; SAF, sodium alginate film; ODLRRP, oven-dried lotus rhizome root powder.

The TPCs, DPPH, FICA, and FRPA of SAFs added with different amounts of ODLRRP are shown in Figure 1. The TPCs of SAFs tended to increase with the addition of ODLRRP. SAFs added with 2% ODLRRP (SAFO3) had the highest values of TPCs (p<0.05), which was consistent with the results of Yang et al [11], who observed that lotus rhizome contained TPCs known to be positively correlated with antioxidant activity. In addition, Zhao et al [10] compared the antioxidant activity and functional components of lotus roots from different growing regions and showed a correlation between the TPCs in lotus rhizomes and antioxidant activity.

When the film was diluted to 0.5% solution, the DPPH level remained constant regardless of the concentration of ODLRRP. The DPPH values of SAF treated with ODLRRP increased at 1% or 2% concentration compared with those of SAF without ODLRRP (p<0.05). Nur Hanani et al [22] reported that the DPPH of the fish gelatin film increased depending on the concentration of pomegranate peel powder, which was rich in phenolic compounds and anthocyanins, contributing to its potent antioxidant activity. Tsuruta et al [23] reported that the high levels of polyphenolic compounds in LRR contributed to its antioxidant and anti-inflammatory activities. Therefore, in this study, the addition of ODLRRP to SAFs might increase the DPPH due to the increased levels of phenolic compounds.

The FICA of SAFs treated with 2% ODLRRP was increased in the 0.5% film solution compared with that in the control sample (p<0.05). The FICA of SAF containing more than 0.5% ODLRRP showed a higher value than the control sample when the concentration of the film solution was higher than 1%. The reduction of Fe^2+^ protected food from oxidative damage-induced free radicals and lipid peroxidation [24]. Thus, the increase in FICA increased the antioxidant activity. Lee et al [24] reported that treatments with *Moringa oleifera* Lam. leaf extract containing polyphenolic compounds increased the FICA of puffer fish skin gelatin film.

In addition, the FRPA of SAF treated with 2% ODLRRP in the 2% film solution was higher than that of the control sample (p<0.05). Our results of FRPA were similar to those reported by Chentir et al [25] who determined that biofunctional films based on bovine gelatin added with 6.25% or 12.5% phycocyanin showed higher FICA and FRPA than films without phycocyanin. Therefore, addition levels higher than 1% ODLRRP increased the antioxidant activities of SAF, which might improve the shelf-life of meat products when the film is used as an outer packaging or coating.

### Physicochemical properties, antioxidant activities, and antimicrobial activities of different sodium alginate films treated with different amounts of oven-dried lotus rhizome root powder during storage

#### pH and color values

The results of the pH and color measurements of different SAFs are shown in Table 2. The addition of ODLRRP did not change the pH values. During storage, the pH values of different SAFs also remained unchanged. However, the L*-value of SAFs decreased with the addition of ODLRRP (p<0.05). It was further decreased with the increased level of addition of ODLRRP. The a*- and b*-values of SAF increased with the addition of ODLRRP in a dose-dependent manner. Color differences among the SAFs listed in Table 1 were mainly attributed to the addition of the ODLRRP because ODLRRP underwent the Maillard reaction or other browning reactions during drying. When different concentrations of ODLRRP were added to the SAF, they affected the color of the SAF as well. During storage, the L*- and a*-values of SAF tended to decrease, whereas the b*-value increased, probably due to the oxidative activity and the yellow pigment of ODLRRP itself, which was reported in a previous study by Qiu and Chin [26]. Lipid oxidation produces peroxides that promote pigment oxidation and affect pigment stability, leading to color fading [27].

#### TBARS2-Thiobarbituric acid-reactive substances, volatile basic nitrogen, microbial counts, and increase in water

The TBARS, VBN, microbial counts, and IW of SAFs with different concentrations of ODLRRP are summarized in Table 3. The TBARS values of SAFs added with ODLRRP decreased compared to those of SAFs alone (p<0.05). SAFs added with 1% ODLRRP (SAFO2) and SAFO3 reduced the TBARS values more than SAFs added with 0.5% ODLRRP (SAFO1). Therefore, SAFO2 and SAFO3 had higher antioxidant activities than SAFO1 or the control. This might be due to the higher TPCs and antioxidant activity of 1% and 2% ODLRRP than the 0.5% ODLRRP, which was consistent with the results of Figure 1. Because TPCs could generate hydrogen atoms to interrupt the free radical oxidation chain, lipid oxidation was reduced in beef patties added with tea catechins and vitamin C compared to control samples [27]. In addition, TBARS tended to increase due to spoilage during storage, and the difference appeared on day 10. No differences in VBN values were observed for any film added with or without ODLRRP, and the storage period did not alter the VBN.

The addition of ODLRRP decreased the TBC of SAFs (Table 3). The higher the concentration of ODLRRP was, the greater the decrease in the TBC of SAFs was. The microbial counts of *Enterobacteriaceae* of SAFs added with ODLRRP showed a similar trend as the TBC. In this study, the reduction in microbial counts of SAFs was due to the addition of ODLRRP, which was supported by Chakravorty et al [28], who reported that *Nulembo nucifera* rhizome extract had antibacterial activity against Gram-positive and Gram-negative bacteria. Due to the antimicrobial phenolic substances, such as phenolic acids, flavonoids, and tannins in ODLRRP, the TPCs of SAFs increased with the level of ODLRRP, thereby reducing the microbial population of SAFs even more.

The IW values of SAFO1 after water absorption from pork patties remained unchanged, whereas those of SAFO2 were reduced compared with those of SAFs without ODLRRP (Table 3). However, Sharma et al [29] reported that the addition of *Rubia cordifolia* increased the MC of the bioactive edible film by altering its hydrophobicity or hydrophilicity. In the present study, the decrease in IW might be attributed to the higher water content and lower EWC in SAFO2 and SAFO3 than in SAF without ODLRRP, thereby affecting their water permeability.

### Physicochemical properties, antioxidant activities, and antimicrobial activities of pork patties wrapped with various sodium alginate films added with different levels of oven-dried lotus rhizome root powder during storage

#### pH and color values

The results of the pH and color measurements of pork patties wrapped with SAFs containing different amounts of ODLRRP are shown in Table 4. It was found that the pH values of pork patties were not altered by the SAFs wrapping and increased on day 7 of storage. Similarly, a study by Ghaderi-Ghahfarokhi et al [30] reported that the accumulation of volatile alkaline nitrogen compounds due to microbial spoilage increased the pH values of beef patties during storage.

Only patties wrapped with SAFO3 showed decreased L*-value and increased b*-value, which was explained by the fact that SAFO3 had the lowest L*-value and the highest b*-value (Table 2). During storage, the a*-value of pork patties tended to decrease from day 10, whereas the b*-value of pork patties increased on day 10. These results were consistent with a study by Qiu and Chin [26], who reported changes in color during storage, including decreased redness and increased yellowness of pork patties due to lipid oxidation.

#### 2-Thiobarbituric acid-reactive substances, volatile basic nitrogen, microbial counts, and weight loss

The TBARS, VBN, microbial counts, and WL of pork patties wrapped with SAFs added with different levels of ODLRRP are shown in Table 5. The TBARS values of pork patties were not changed by film wrapping, despite differences in the TBARS values of SAFs added with different amounts of ODLRRP (Table 3), whereas they gradually increased during storage. Soni et al [31] reported that chicken patties wrapped with edible films containing essential oils reduced the TBARS values compared to control samples due to the antioxidant activity of essential oils. In addition, the thickness of each SAF listed in Table 1 did not vary, which might contribute to the constant oxygen permeability of different SAFs. Thus, the TBARS values of pork patties wrapped with different SAFs remained unchanged. In addition, it should be noted that ODLRRP might need to be made into an extract to increase its antioxidant activity and thus reduce the TBARS values, although the antioxidant activity of the SAFs was affected by the addition of ODLRRP.

The VBN values of pork patties were reduced by wrapping them with SAF. The SAFs added with ODLRRP decreased the VBN values of pork patties in a dose-dependent manner. The total VBN level might be used to measure protein and amine degradation, thus indicating the freshness of meat [32]. Our results indicated that SAF-wrapped patties inhibited the degradation of protein or amine in pork patties. Among them, ODLRRP increased the inhibition of degradation. Therefore, SAFs added with ODLRRP ensured the freshness of pork patties by lowering the VBN. Raeisi et al [33] reported that sodium alginate coating reduced the total VBN value, indicating that it inhibited protein decomposition. In addition, Yang et al [11] reported that lotus root oil possessed antioxidant activity, which reduced the VBN value of pork patties. During storage, the VBN increased, and a difference in the increase of VBN was found on day 7 and 14, respectively.

Pork patties wrapped with SAFs added with 1% or 2% ODLRRP showed decreases in TBC, consistent with the high antibacterial activity of SAFO2 or SAFO3 (Table 3). The composition of sodium alginate and ODLRRP in the SAF and the low permeability of SAFs were the main factors contributing to the reduction in the number of bacteria. In a related study, Ham et al [34] reported that sodium alginate-carboxymethyl cellulose film treated with cinnamon essential oil exhibited good antibacterial properties, such as resistance to *Escherichia coli* and *Staphylococcus aureus*, and that an increase in the concentration of cinnamon essential oil enhanced the antibacterial ability. In the present study, the increase in the concentration of ODLRRP in SAFs also reduced the TBC, which might be due to the antibacterial ability of lotus roots, as reported by Chakravorty et al [28]. Furthermore, the permeability of different SAFs might not be affected by the addition of ODLRRP because the thicknesses were the same among different SAFs. During storage, the TBC of pork patties was detected from day 7, whereas the *Enterobacteriaceae* count was detected from day 10. Subsequently, the TBC and *Enterobacteriaceae* count of pork patties gradually increased until day 14. Smolander et al [35] reported that *Enterobacteriaceae* was a family of facultative anaerobic Gram-negative bacteria indicating hygienic and environmental conditions with a close relationship with sensory odor. Therefore, patties might have deteriorated and produced an off-odor from day 10 of storage, the day *Enterobacteriaceae* were detected.

Although pork patties wrapped with SAFs did not affect the WL, the WL tended to increase during storage and showed a significant difference by day 10. During storage, patties became dry and hard, which might have increased the WL of patties.

#### Proximate compositions

The proximate compositions of pork patties are shown in Table 6. The protein content of pork patties was increased by SAF wraps containing ODLRRP. However, the protein content of pork patties wrapped with SAFs alone (not incorporated with ODLRRP) was not changed. Only wrapping with SAFs added with 0.5% ODLRRP reduced the fat contents of pork patties. At the same time, SAFs added with 2% ODLRRP reduced the MC of pork patties compared with SAFs added with 0.5% ODLRRP or SAFs alone. The decrease in MC (%) of pork patties wrapped with SAFs containing 2% ODLRRP might be due to the ability of SAFs to absorb moisture from pork patties during storage. Ham et al [34] reported that the addition of LRRP increased the MC of cooked sausages due to the high water absorption of dietary fiber in LRRP. Fernández-Ginés et al [36] also reported that bologna sausages added with 2.5% or 5.0% lemon albedo exhibited an increased MC due to the water-holding capacity of lemon albedo. These two papers could explain the observation that SAFs absorbed water from the patties and could retain water to avoid loss in this study. Because the SAFs absorbed water from patties, the protein or fat contents (%) of patties increased relatively. The addition of 2% ODLRRP increased the water absorption of SAFs, resulting in decreased MC of patties. During storage, the protein and fat contents (%) of pork patties increased on day 14, followed by a decrease in MC (%).

## CONCLUSION

The addition of ODLRRP reduced the transparency, EWC, L*, and pH values of SAFs. However, it increased the a* value, b* alue, and antioxidant and antimicrobial activities of SAFs. Patties wrapped with SAFs containing ODLRRP exhibited decreased VBN, microbial counts, and MC (%) but increased protein content (%). Pork patties wrapped with SAFs containing 1% or 2% ODLRRP showed similar antibacterial activity and VBN values. Compared with SAFs containing 1% ODLRRP, SAFs containing 2% ODLRRP were darker in color and had reduced transparency. Based on these results, SAFs containing 1% ODLRRP could be used as a bioactive film to wrap pork patties for the extension of the shelf-life during storage. Although SAFs containing ODLRRP had a positive effect on the shelf-life of the patties, consideration should be given to reducing the moisture in the patties due to the absorption of water by SAFs in order to avoid economic losses. Future research should avoid the water absorption capacity of SAFs as much as possible and endeavor to create healthier, tempting, and edible SAFs for customer choice and preference.

## Figures and Tables

**Table 1:** Properties of SAF by the level of incorporated with ODLRRP

Parameters	SAF	SAFO1	SAFO2	SAFO3	F	Standard error
pH	5.93±0.02^a^	5.87±0.02^b^	5.78±0.01^c^	5.77±0.01^c^	69.07	0.02
WI (%)	1.90±0.07^a^	2.27±0.61^a^	1.83±0.08^a^	1.93±0.13^a^	1.17	0.09
L*	87.10±0.56^a^	81.00±0.00^b^	75.87±0.50^c^	66.63±2.14^d^	175.61	2.28
a*	1.83±0.06^c^	1.93±0.21^c^	3.50±0.35^b^	6.57±0.75^a^	80.16	0.59
b*	−5.47±0.06^d^	6.90±0.26^c^	14.93±0.57^b^	24.20±0.26^a^	4,069.07	3.29
ΔE*	11.67±0.50^d^	24.10±0.17^c^	33.43±0.72^b^	46.60±1.65^a^	743.73	3.86
Chroma (C)*	5.77±0.06^d^	7.17±0.25^c^	15.40±0.62^b^	25.10±0.44^a^	1,472.20	2.33
Thickness (mm)	0.0152±0.0009^a^	0.0155±0.0010^a^	0.0160±0.0012^a^	0.0164±0.0010^a^	0.72	0.0003
Transparency	12.94±4.28^d^	40.57±6.66^c^	60.90±4.91^b^	75.24±5.35^a^	76.00	7.18
MC (%)	18.23±1.08^b^	21.13±0.65^a^	21.37±0.45^a^	20.67±1.91^a^	4.57	0.47
WS (%)	70.88±6.77^a^	70.24±8.83^a^	64.67±11.18^a^	63.00±13.14^a^	0.45	2.73
EWC (%)	10.90±2.17^a^	8.69±1.87^ab^	7.53±1.35^b^	8.03±0.64^ab^	2.55	0.56

a–d Mean with different superscripts in the different treatments are different (p<0.05).

ODLRRP, oven-dried lotus rhizome root powder (particle size≤150 μm); SAF, sodium alginate films; SAFO1, sodium alginate films with 0.5% ODLRRP; SAFO2, sodium alginate films with 1% ODLRRP; SAFO3, sodium alginate films with 2% ODLRRP; WI, water increase of film after cross-link; L*, lightness; a*, redness b*, yellowness; MC, moisture contents; WS, water solubility; EWC, equilibrium water content.

**Table 2:** pH and color values (L*, a*, b*) of SAF incorporated with different levels of ODLRRP

Item	pH	L*	a*	b*
TRT	NS	^**^	^**^	^**^
DAY	NS	^**^	^**^	^**^
TRT×DAY	NS	NS	NS	NS
SAF	6.31±0.28^a^	83.5±2.07^a^	1.47±0.59^d^	−4.51±2.17^d^
SAFO1	6.26±0.16^a^	78.3±1.37^b^	2.14±0.47^c^	5.85±2.09^c^
SAFO2	6.22±0.12^a^	73.2±4.82^c^	3.15±0.73^b^	12.96±1.99^b^
SAFO3	6.15±0.09^a^	66.6±6.12^d^	5.35±0.75^a^	20.38±1.98^a^
Storage days				
0	6.22±0.21^A^	77.5±5.59^A^	3.43±1.57^A^	7.26±4.41^C^
3	6.25±0.22^A^	77.4±5.61^A^	3.40±1.70^A^	7.93±4.61^BC^
7	6.30±0.19^A^	75.6±7.29^AB^	3.11±1.59^A^	8.32±5.80^BC^
10	6.25±0.15^A^	74.3±9.27^AB^	2.98±1.72^A^	9.40±6.26^AB^
14	6.16±0.12^A^	72.2±8.60^B^	2.23±1.40^B^	10.46±6.59^A^

A–C Means with different superscripts in the same column are different (p<0.05).

a–d Means with different superscripts in the same column are different (p<0.05).

** p<0.01.

L*, lightness; a*, redness b*, yellowness.SAF, sodium alginate films; ODLRRP, oven-dried lotus rhizome root powder (particle size≤150 μm); TRT, treatments; NS, no significant difference; SAFO1, sodium alginate films with 0.5% ODLRRP; SAFO2, sodium alginate films with 1% ODLRRP; SAFO3, sodium alginate films with 2% ODLRRP.

**Table 3:** TBARS, VBN, microbial counts, and IW of SAF incorporated with different levels of ODLRRP

Item	TBARS (mg malondialdehyde/kg)	VBN (mg %)	TBs (Log cfu/g)	EBs (Log cfu/g)	IW (%)
TRT	^*^	NS	^**^	^**^	^*^
DAY	^**^	NS	^**^	^**^	NS
TRT×DAY	NS	NS	NS	^**^	NS
SAF	2.20±0.80^a^	6.44±2.38^a^	4.05±0.54^a^	2.07±1.78^a^	27.49±23.36^a^
SAFO1	1.95±0.76^ab^	5.96±2.16^a^	3.88±0.46^ab^	1.92±1.66^b^	22.30±24.89^a^
SAFO2	1.78±0.66^b^	5.37±1.63^a^	3.75±0.47^b^	1.30±1.67^c^	3.68±40.08^ab^
SAFO3	1.64±0.59^b^	5.12±1.29^a^	3.45±0.51^c^	1.18±1.52^c^	−5.26±41.27^b^
Storage days					
0	1.36±0.37^C^	4.87±2.04^A^	3.19±0.32^E^	<2.00±0.00^D^	18.03±18.71^A^
3	1.53±0.46^C^	5.14±1.91^A^	3.46±0.34^D^	<2.00±0.00^D^	23.58±19.06^A^
7	1.61±0.44^C^	5.62±1.56^A^	3.81±0.34^C^	1.40±1.48^C^	23.57±18.52^A^
10	2.14±0.41^B^	6.22±2.07^A^	4.09±0.32^B^	3.10±0.40^B^	2.31±49.37^A^
14	2.83±0.71^A^	6.77±1.74^A^	4.37±0.31^A^	3.58±0.33^A^	−7.23±48.34^A^

A–E Means with different superscripts in the same column are different (p<0.05).

a–c Means with different superscripts in the same column are different (p<0.05).

* p<0.05,

** p<0.01.

TBARS, 2-thiobarbituric acid-reactive substances; VBN, volatile basic nitrogen; IW, increase in water; SAF, sodium alginate films; ODLRRP, oven-dried lotus rhizome root powder (particle size≤150 μm); TBs, total bacteria counts; EBs, *Enterobacteriaceae* counts; TRT, treatments; NS, no significant difference; SAFO1, sodium alginate films with 0.5% ODLRRP; SAFO2, sodium alginate films with 1% ODLRRP; SAFO3, sodium alginate films with 2% ODLRRP.

**Table 4:** pH and color values (L*, a*, b*) of pork patties wrapped in SAF incorporated with different ODLRRP

Treatments	pH	L*	a*	b*
TRT	NS	^*^	NS	^*^
DAY	^**^	NS	^**^	NS
TRT×DAY	NS	NS	NS	NS
PCTL	5.61±0.06^a^	56.17±2.19^a^	5.63±2.69^a^	10.89±1.38^b^
PSAF	5.61±0.06^a^	55.43±1.93^a^	5.30±2.96^a^	10.71±1.44^b^
PSAFO1	5.60±0.06^a^	55.91±2.15^a^	5.40±2.56^a^	10.86±1.06^b^
PSAFO2	5.61±0.06^a^	55.15±2.57^a^	5.59±2.22^a^	11.61±0.93^ab^
PSAFO3	5.62±0.05^a^	53.29±2.31^b^	5.93±1.88^a^	11.97±0.72^a^
Storage days				
0	5.58±0.02^B^	54.87±2.60^A^	7.18±1.79^A^	10.58±1.37^B^
3	5.55±0.01^B^	55.25±1.44^A^	6.99±1.86^A^	11.07±1.11^AB^
7	5.64±0.07^A^	55.05±1.68^A^	5.63±2.25^AB^	11.27±1.41^AB^
10	5.65±0.03^A^	55.41±2.69^A^	4.60±1.89^BC^	11.75±1.12^A^
14	5.64±0.05^A^	55.37±3.39^A^	3.44±2.26^C^	11.37±0.79^AB^

A–C Means with different superscripts in the same column are different (p<0.05).

a,b Means with different superscripts in the same column are different (p<0.05).

* p<0.05,

** p<0.01.

L*, lightness; a*, redness b*, yellowness; SAF, sodium alginate films; ODLRRP, oven-dried lotus rhizome root powder (particle size≤150 μm); TRT, treatments; NS, no significant difference; PCTL, patties without film wrapping; PSAF, patties wrapped in sodium alginate films; PSAFO1, patties wrapped in sodium alginate films containing 0.5% ODLRRP; PSAFO2, patties wrapped in sodium alginate films containing 1% ODLRRP; PSAFO3, patties wrapped in sodium alginate films containing 2% ODLRRP.

**Table 5:** TBARS, VBN, microbial counts and WL of pork patties wrapped in SAF incorporated with different levels of ODLRRP

Treatments	TBARS (mg malondialdehyde/kg)	VBN (mg%)	TBs (Log CFU/g)	EBs (Log CFU /g)	WL (%)
TRT	NS	^**^	^**^	NS	NS
DAY	^**^	^**^	^**^	^**^	^**^
TRT×DAY	NS	NS	^**^	NS	NS
PCTL	1.68±1.27^a^	8.00±1.38^a^	2.00±1.74^a^	1.27±1.63^a^	5.47±4.82^a^
PSAF	1.74±1.18^a^	7.75±1.28^ab^	1.97±1.72^a^	1.23±1.57^a^	7.37±4.86^a^
PSAFO1	1.50±1.07^a^	7.31±1.33^bc^	1.86±1.63^a^	1.18±1.51^a^	7.35±8.18^a^
PSAFO2	1.31±0.85^a^	6.93±1.31^cd^	1.33±1.72^b^	1.23±1.58^a^	7.83±8.83^a^
PSAFO3	1.24±0.86^a^	6.54±1.15^d^	1.27±1.64^b^	1.19±1.54^a^	8.17±9.66^a^
Storage days					
0	0.55±0.30^D^	5.94±0.82^C^	<2.00±0.00^D^	<2.00±0.00^C^	2.39±2.29^C^
3	0.94±0.64^CD^	6.50±1.05^C^	<2.00±0.00^D^	<2.00±0.00^C^	3.92±1.79^BC^
7	1.34±0.81^BC^	7.37±1.03^B^	1.59±1.36^C^	<2.00±0.00^C^	4.74±1.93^BC^
10	1.79±0.67^B^	7.81±0.85^B^	3.24±0.45^B^	2.82±0.32^B^	8.41±3.20^B^
14	2.85±0.92^A^	8.91±0.74^A^	3.60±0.39^A^	3.29±0.26^A^	16.75±11.21^A^

A–D Means with different superscripts in the same column are significantly different (p<0.05).

a–d Means with different superscripts in the same column are significantly different (p<0.05).

* p<0.05,

** p<0.01.

TBARS, 2-thiobarbituric acid-reactive substances; VBN, volatile basic nitrogen; WL, weight loss; SAF, sodium alginate films; ODLRRP, oven-dried lotus rhizome root powder (particle size≤150 μm); TBs, total bacteria counts; EBs, *Enterobacteriaceae* counts; TRT, treatments; NS, no significant difference, PCTL, patties without film wrapping; PSAF, patties wrapped in sodium alginate films; PSAFO1, patties wrapped in sodium alginate films containing 0.5% ODLRRP; PSAFO2, patties wrapped in sodium alginate films containing 1% ODLRRP; PSAFO3, patties wrapped in sodium alginate films containing 2% ODLRRP.

**Table 6:** Proximate composition of pork patties wrapped in SAF incorporated with different levels of ODLRRP

Treatments	Protein	Fat	Moisture
TRT	^**^	^*^	NS
DAY	^**^	^**^	^**^
TRT×DAY	NS	NS	NS
PCTL	18.69±1.77^b^	18.92±2.32^a^	57.37±4.33^ab^
PSAF	18.91±1.20^b^	17.76±0.91^ab^	58.90±1.71^a^
PSAFO1	20.41±2.80^a^	17.02±1.24^b^	59.54±1.50^a^
PSAFO2	20.43±2.77^a^	18.30±1.50^a^	55.99±7.00^ab^
PSAFO3	20.82±3.22^a^	18.41±1.86^a^	53.99±9.74^b^
Storage days			
0	18.11±1.14^B^	17.53±0.46^B^	59.93±0.69^A^
7	18.95±0.82^B^	17.47±0.93^B^	58.51±1.02^A^
14	22.50±2.50^A^	19.25±2.40^A^	53.03±8.89^B^

A,B Means with different superscripts in the same column are significantly different (p<0.05).

a,b Means with different superscripts in the same column are significantly different (p<0.05).

* p<0.05,

** p<0.01.

SAF, sodium alginate films; ODLRRP, oven-dried lotus rhizome root powder (particle size≤150 μm); TRT, treatments; NS, no significant difference; PCTL, patties without film wrapping; PSAF, patties wrapped in sodium alginate films; PSAFO1, patties wrapped in sodium alginate films containing 0.5% ODLRRP; PSAFO2, patties wrapped in sodium alginate films containing 1% ODLRRP; PSAFO3, patties wrapped in sodium alginate films containing 2% ODLRRP.
